# “Flash” preparation of strongly coupled metal nanoparticle clusters with sub-nm gaps by Ag^+^ soldering: toward effective plasmonic tuning of solution-assembled nanomaterials†
†Electronic supplementary information (ESI) available: Experimental details, a video clip of the soldering process, and extra supporting data. See DOI: 10.1039/c6sc01407k


**DOI:** 10.1039/c6sc01407k

**Published:** 2016-05-04

**Authors:** Miao Liu, Lingling Fang, Yulin Li, Ming Gong, An Xu, Zhaoxiang Deng

**Affiliations:** a CAS Key Laboratory of Soft Matter Chemistry & Collaborative Innovation Center of Suzhou Nano Science and Technology , Department of Chemistry , University of Science and Technology of China , Hefei , Anhui 230026 , China . Email: zhxdeng@ustc.edu.cn; b Engineering and Materials Science Experiment Center , University of Science and Technology of China , Hefei , Anhui 230027 , China; c Key Laboratory of Ion Beam Bioengineering , Hefei Institutes of Physical Science , Chinese Academy of Sciences , Hefei , Anhui 230031 , China

## Abstract

Noble metal nanoparticle oligomers are important in applications including plasmonics, catalysis, and molecular sensing. These nanostructural units featuring abundant inter-particle junctions are helpful for a physical/chemical understanding of structure-activity relationships of self-assembled metamaterials. A simple, rapid, and potentially general strategy for the preparation of monodisperse nanoparticle clusters in a homogeneous solution is highly desired for fundamental research toward liquid metamaterials and chemical/biological applications, but this is however very challenging. Here we report an Ag^+^ soldering strategy to prepare strongly coupled plasmonic (Au) and catalytic (Pt, Au@Pd (Au core with a Pd shell)) nanoparticle clusters almost instantly (<1 min) in a solution without special synthetic efforts, complicated surface decorations, or structure-directing templates. The resulting clusters are isolatable by agarose gel electrophoresis, resulting in mechanically stable products in high purity. The optical extinctions of Au nanodimers (the simplest and most basic form of a coupled structure) exhibit prominent longitudinal plasmonic coupling for nanoparticles down to 13.3 nm in diameter. Theoretical simulations attribute the strong coupling to the existence of a sub-nm gap (*c.a.* 0.76 nm) between soldered particles, suggesting an ideal (stable, soluble, monodisperse, and weakly passivated) substrate for surface enhanced Raman scattering (SERS) applications.

## Abstract 2

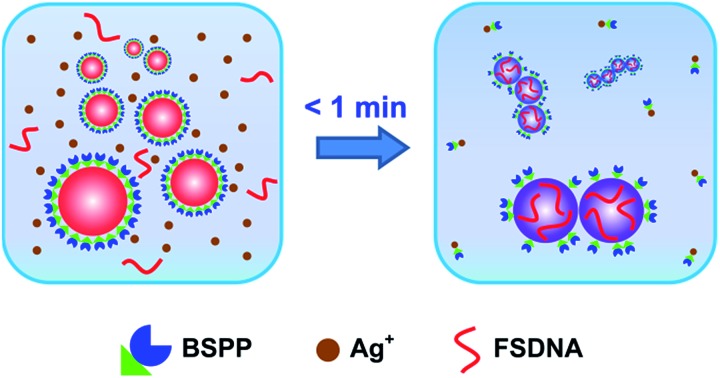
An Ag^+^ soldering strategy is developed to achieve strongly coupled nanoparticle clusters for plasmonic and surface enhanced Raman scattering applications.

## Introduction

Self-assembly represents a key route to nanoparticle-based metamaterials with appealing properties due to their tailorable ensemble behavior and physical/chemical coupling.^1^,^2^ In particular, noble metal-based nanoparticle clusters are important for plasmonic optics,^3^–^5^ catalysis,^6^–^8^ molecular sensing,^9^,^10^ and metafluids^11^,^12^ (liquids with negative indexes). Although DNA is a very useful tool for programmable material assembly,^13^–^15^ the inter-particle distance in a DNA-assembled nanoparticle cluster often falls outside the range of a strong physical/chemical coupling toward effective structural and functional tuning.^2^,^4^,^16^,^17^ We have recently developed an Ag^+^ soldering process to address this dilemma.^18^ However, the involvement of nanoparticles with increased sizes will cause difficulty in making DNA–nanoparticle conjugates with specifiable DNA valences^14^ for accurate structure programming. Other foreseeable difficulties include lowered colloidal stability and decreased DNA hybridization efficiency. Nanostructures built from large nanoparticles are very important due to their significantly boosted interaction with light.^19^ A method that does not rely on DNA programming to form well-defined and strongly coupled nanostructures is therefore highly expected. This process will also be suitable for large scale productions.

To obtain high purity nanoparticle clusters (such as dimers), a physical interface is often utilized to provide a spatial restriction^20^ for asymmetric ligand functionalization and sterically confined nanoparticle assembly.^21^–^29^ Other strategies include controllable heterogeneous nucleation on a seed particle,^30^,^31^ centrifugation-induced electrical polarization^32^ of nanoparticles, and the use of a two-dimensional (2D) dithiol crystal for nanoparticle attachment so that dimers are formed after crystal disassembly.^33^

Despite this progress, the pursuit toward a fully solution-based method to make discrete nanoparticle clusters is still challenging in the absence of a structure-directing template (such as DNA).^34^,^35^ This is mainly due to the lack of an easy way to prevent uncontrollable nanoparticle aggregation. On the one side, a strong enough inter-particle interaction is needed to form a stable assembly; on the other side, such a force should be weak enough to avoid large aggregates. Although a very careful choice of nanoparticle and linker molecule concentrations sometimes results in enriched dimers, it is very hard to achieve high product purity (*e.g.* >90%) and to assemble a cluster containing more than two particles.^36^–^38^ Previous work has also used specially synthesized bi- or multi-functional organic thiol linkers with rigid arms of controlled length for the assembly of gold and silver nanoparticle clusters.^39^,^40^ In some other attempts, polymer ligands have been decorated on nanoparticles so that nanoparticle clusterization can take place *via* hydrophobic interactions.^41^ Emulsion droplet evaporation is an important method to generate nanoparticle clusters with high coordination numbers.^42^ Chen *et al.* have developed a general and efficient process to make nanoparticle clusters *via* polymer micelle entrapping.^43^,^44^

Some existing strategies could only produce strongly-coupled nanoparticle clusters on a substrate (dried form),^4^,^5^,^45^ which are incompatible with chemical/biological applications (a solution environment is often required) as well as pursuits toward novel liquid materials (*e.g.* metafluids).^11^,^12^ Also, the available methods usually need the help of thiol ligands^5^,^41^,^46^ and thus may cause a permanent blocking of nanoparticles' surface activities. Moreover, special care is necessary to avoid the over-crosslinking of nanoparticles into bulky precipitates, which increases experiment complexity and time. Methods based on DNA assembly often rely on a dehydration process^4^,^45^ or seeded overgrowth^9^ to achieve strong inter-particle coupling, which may not be suitable for producing stable and monodisperse structures in a solution.

**Fig. 1:**
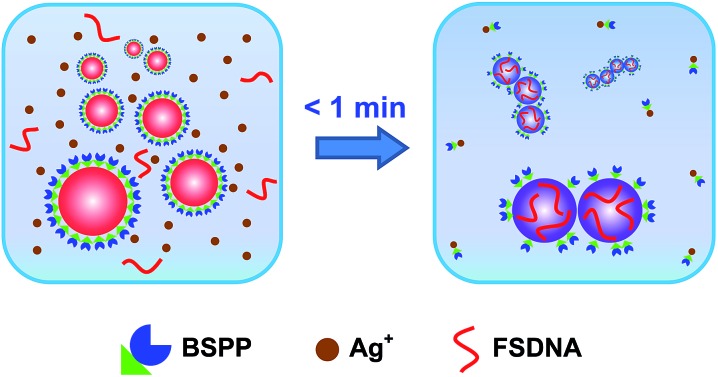
Schematic illustration of an Ag^+^ soldering-based “flash” assembly of highly stable nanoparticle clusters with very strong inter-particle coupling. BSPP and FSDNA depict bis(*p*-sulfonatophenyl) phenyl phosphine dipotassium salt and mechanically shortened fish sperm DNA, respectively.

In this work, we report a simple, rapid, and easily controllable method to make strongly coupled nanoparticle clusters with excellent stability, high purity, and minimum surface passivation (without completely blocking the surface) by virtue of a solution-based Ag^+^ soldering process (Fig. 1). Here, the bis(*p*-sulfonatophenyl) phenyl phosphine (BSPP) ligand plays a dual role: to stabilize metal (Au, Pt, and Au@Pd (Au core with a Pd shell)) nanoparticles and to bond with Ag^+^. We assume that Ag^+^ is able to strip off some BSPP ligands due to the well-known Ag^+^ affinity of BSPP.^47^,^48^ This causes quick destabilization of the nanoparticles to form clustered structures. Immediately, FSDNA (mechanically shortened fish sperm DNA) gets a chance to interact with the partially exposed (due to BSPP stripping) nanoparticle surface,^49^,^50^ and terminates further clusterizations due to increased steric repulsion. X-ray photoelectron spectroscopy (XPS) analyses provided evidence for the BSPP stripping and the DNA adsorption (Fig. S1–S3†). A decrease of BSPP content (based on the XPS signals of trivalent P and sulfonic S) and an increase of the P and N signals from the phosphate backbone and nucleobases of DNA were clearly judged for the soldered structures.

Our method has several advantages: (1) the reaction is finished instantly (<1 min, see the ESI video clip†), which can be easily controlled by Ag^+^; (2) it is a one-pot solution process capable of producing highly water-dispersible clusters; (3) all chemicals are commercially available such that no synthetic efforts are needed; (4) the soldered structures appear as sharp bands during agarose gel electrophoresis, facilitating a quick and high resolution gel isolation; and (5) such a process is adaptable to different nanomaterials.

## Results and discussion

**Fig. 2:**
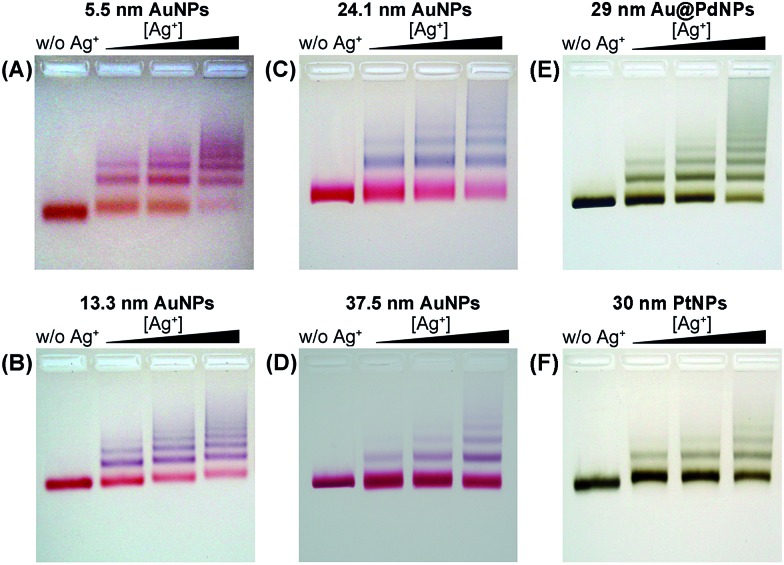
Agarose gel electrophoresis of AuNPs (A–D), Au@PdNPs (E), and PtNPs (F) after Ag^+^ soldering. Gel bands in the same vertical lanes correspond to discrete clusters containing increased numbers (1, 2, 3, and so on) of nanoparticles.

**Fig. 3:**
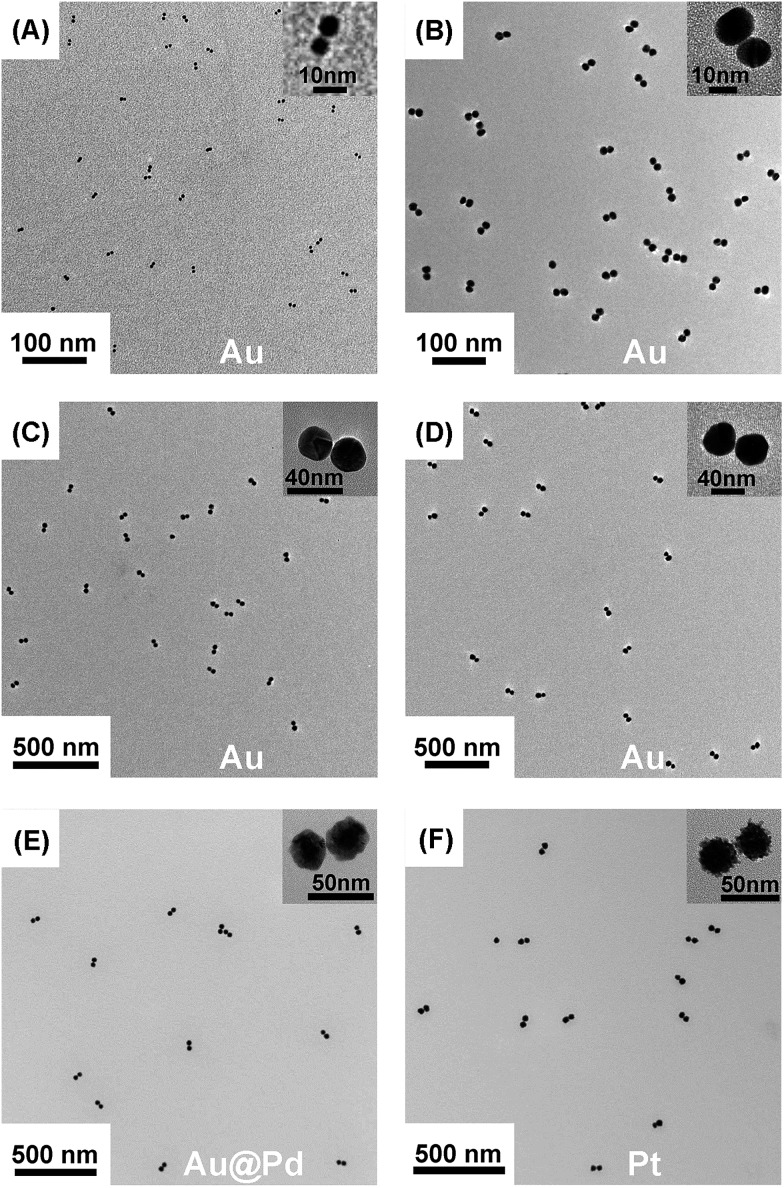
TEM images of gel-isolated dimeric clusters formed by AuNPs (A–D), Au@PdNPs (E), and PtNPs (F). Panels A–D correspond to AuNPs with diameters of 5.5 nm (A), 13.3 nm (B), 24.1 nm (C), and 37.5 nm (D). The diameters of Au@Pd and Pt nanoparticles were 29 nm and 30 nm, respectively. Statistical analysis based on TEM data revealed 95%, 98%, 94%, 93%, 91%, and 97% yields for the gel-purified 5.5–37.5 nm AuNP dimers and Au@PdNP and PtNP dimers, respectively (Table S1†).

We employed gold nanoparticles (AuNPs) with diameters of 5.5, 13.3, 24.1, and 37.5 nm, as well as Au@Pd core–shell nanostructures and Pt nanoparticles, to form discrete cluster structures. Note that Ag^+^ soldering is different from a regular colloidal destabilization process where nanoparticles keep aggregating and are precipitated. The Ag^+^-controlled process reached a steady self-limiting phase immediately upon adding a certain amount of Ag^+^ and FSDNA (see ESI† for experimental details). Fig. 2 shows well-separated product bands with clear color transitions for AuNPs (Fig. 2A–D) corresponding to increased cluster sizes. In the same lane, each band corresponded to a specific number of nanoparticles soldered together. Considering that dimers are the most basic coupled structures, we focused on dimeric products of different nanoparticles for further characterization. Thanks to a high resolution separation of the dimers by gel electrophoresis (Fig. 2) and the good mechanical stability (see discussions below) of the soldered structures, high purity products were observed by transmission electron microscopy (TEM) (Fig. 3A–D). In addition to AuNP dimers, Fig. S4† shows TEM images and optical extinction data of as-purified trimer and tetramer clusters of the 13.3 nm AuNPs.

Besides AuNPs, it is important to emphasize that this strategy worked similarly well for other types of materials including Au@Pd core–shell structures and superstructured Pt nanoparticles (Fig. 2E, F and Fig. 3E, F), demonstrating the good generality of our method. Note that the 30 nm Pt supraparticles were composed of many fine (4–6 nm) PtNPs, which could still form very stable oligomers *via* Ag^+^ soldering.

The gel-purified AuNP dimers were stored in water for over one month to evaluate their long-term stability. No dissociation or aggregation of the dimers happened, as verified by gel electrophoresis (Fig. S5†). Furthermore, the dimers were subjected to strong sonication *via* a horn-type ultrasonicator. Fig. S6† shows negligible disruption of the 5.5 nm and 13.3 nm AuNP dimers, attesting to their superb mechanical stability. The larger dimers (24.1 and 37.5 nm) exhibited lower but still good stability considering the very harsh sonication treatment.

To exclude the influence of any possibly existing Ag(0) species on the resulting clusters, the dimers were incubated in 10% H_2_O_2_ for a prolonged period of time. This step has been found to be effective in removing metallic Ag *via* oxidative etching.^18^,^51^ The gel electrophoresis and spectral data had no indications of structural or extinction changes of the dimers after the etching (Fig. S7†). Therefore, it is not an essential step to add H_2_O_2_ (or any other chemical etchants) as no sign for the existence of metallic silver was observed.

We found that the soldering was specific to Ag^+^. Other metal ions did not result in stable and well-defined product bands in the gel (Fig. S8†). These metal ions, however, could increase the efficiency of Ag^+^ soldering when co-existing with Ag^+^ at low concentrations due to reduced Coulombic repulsion (Fig. S9†). Also, in the absence of FSDNA, no resolvable product bands were formed (Fig. S10†). Addition of FSDNA to Ag^+^-induced AuNP aggregates could still lead to the formation of discrete nanoparticle clusters during gel separation (Fig. S11†). These phenomena were consistent with the protective role of FSDNA as depicted in Fig. 1, which prevented the formation of uncontrollable AuNP aggregates, and maintained the good stability and high surface charge of the products during gel electrophoresis.^52^ In addition, the surface-adsorbed FSDNA guaranteed the good stability of the dimers in strongly ionic solutions or a saline buffer (Fig. S12 and S13†).

**Fig. 4:**
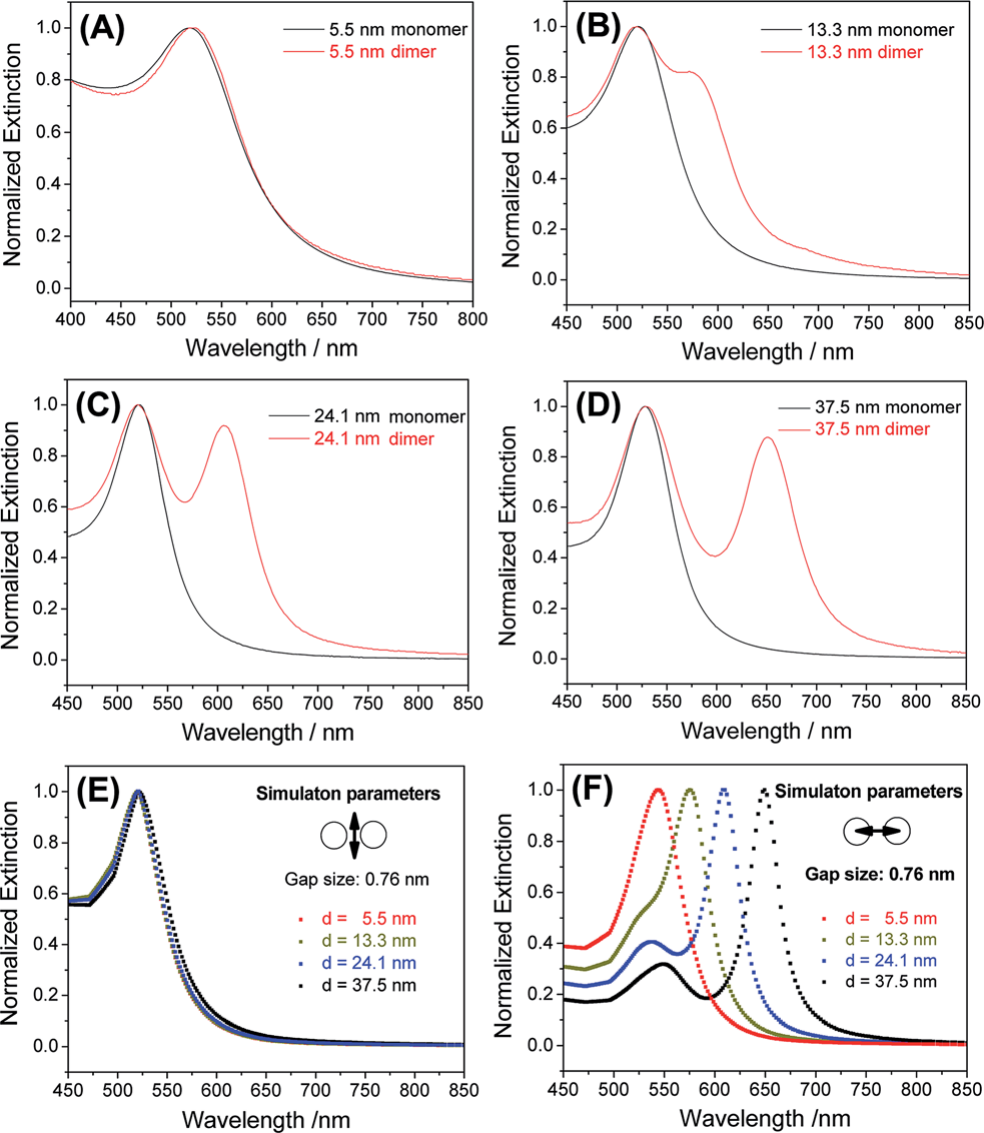
(A–D) Optical extinction spectra of AuNP dimers with different diameters dissolved in aqueous solutions. (E and F) Simulated transverse (E) and longitudinal (F) extinctions of AuNP dimers. An interparticle gap of 0.76 nm was assumed during the simulations. Arrows in panels (E) and (F) indicate the electric field directions of the polarized incident light.

Because a strong inter-particle coupling forms the basis of many important applications, we investigated the plasmonic coupling of the dimers based on their optical extinctions. As shown in Fig. 4A, 5.5 nm AuNP dimers did not show much change of their extinction profile compared to the monomers. With the diameter of AuNPs increased to 13.3 nm, a clearly resolvable shoulder peak representing longitudinal plasmonic coupling started to appear in the dimer's extinction spectrum shown in Fig. 4B. Such a longitudinal resonance happened at a much longer wavelength (574 nm) than the transverse peak (520 nm), implying a strong inter-particle coupling. For even larger AuNPs (24.1 nm and 37.5 nm), completely separated longitudinal resonances were observed at wavelengths of 606 and 651 nm (Fig. 4C and D), respectively. These new peaks were attributable to a strong capacitive plasmonic coupling between two closely spaced AuNPs.^53^,^54^ The rapid red-shift of a longitudinal resonance peak with increased particle size was the result of a reduced *d*/*a* ratio, where *d* and *a* represent the gap size and the nanoparticle radius of a dimer structure, respectively.^55^ In our case, a fixed gap (*d*) could be assumed, while the particle radius (*a*) varied widely. Based on this assumption, we simulated the transverse and longitudinal resonances of Au dimers with a MESME (multiple elastic scattering of multipole expansions) algorithm,^56^,^57^ where the electromagnetic field is decomposed into multipoles around each particle. The calculated transverse and longitudinal extinctions of four different dimers with water as the surrounding media are given in Fig. 4E and F. We found that an inter-particle gap of 0.76 nm (beyond the quantum tunnelling regime of 0.3–0.5 nm (ref. 58 and 59)) achieved the best fitting between the simulated and experimental data for AuNPs of different diameters (13.3 nm and above) (Fig. 4 and S14†). A slight deviation from the experimental extinction data for the 5.5 nm AuNPs could be attributed to a finite size effect which was not modelled by the simulations.^54^ Because the sub-nm gap was very sensitive to an electron beam damage (*e.g.* approaching and coalescence) (Fig. S15†) and a dehydration-induced gap shrinkage,^60^,^61^ such an inter-particle distance obtained by spectral fitting should be more reliable than a TEM measurement.^62^,^63^

In addition to optical extinctions (far field), the dramatically boosted electromagnetic field (near-field) at the sub-nm gap of the Au dimers could be further revealed by surface enhanced Raman scattering (SERS).^62^ Using 4-mercaptopyridine (4-MPy) as a Raman dye, we investigated the AuNP dimers for their SERS activities. Different from commonly used SERS substrates such as surface-coated nanoparticles (in the dry state) or metastable colloidal aggregates, the highly monodisperse, stable, and structurally pure dimers in a homogeneous solution avoided Raman enhancements from anomalous hotspots (uncontrollable aggregates), which is critical to produce highly repeatable SERS signals.^64^

**Fig. 5:**
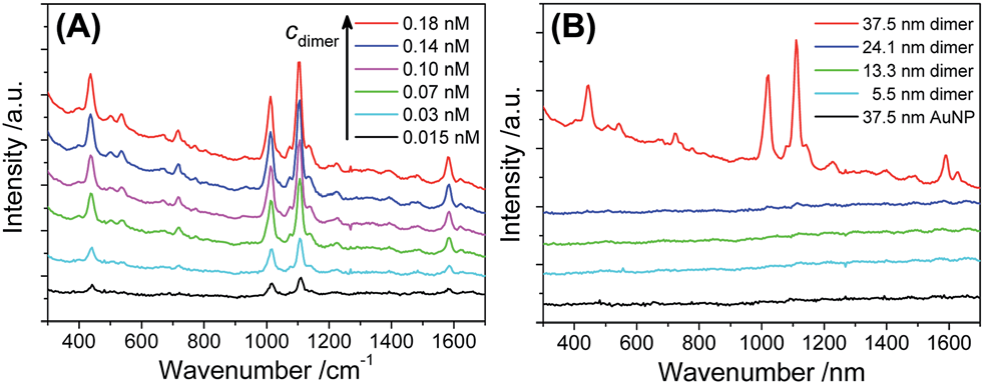
(A) Raman spectra of 4-MPy (5 μM) enhanced by different concentrations of 37.5 nm Au dimers. (B) Raman spectra of 4-MPy (5 μM) measured in the presence of AuNP dimers (0.15 nM) with different diameters, as well as 37.5 nm AuNP monomers (0.3 nM). All spectral curves were vertically shifted for better visibility. Note that the 37.5 nm AuNP dimers were stable in the presence of 4-MPy (5 μM) (Fig. S16†), so that Raman enhancement due to uncontrollable nanoparticle aggregates could be ruled out.

As shown in Fig. 5A, the 37.5 nm AuNP dimers gave a strong Raman scattering. The excitation laser wavelength of 671 nm matched well with the the longitudinal plasmon resonance of the 37.5 nm Au dimers (Fig. 4D). In contrast, a 532 nm laser resulted in very weak Raman scattering of the same sample (Fig. S17†). Dimers of 5.5–24.1 nm AuNPs did not give observable Raman signals mainly due to their smaller light-scattering cross sections (Fig. 5B). Additionally, monomeric 37.5 nm AuNPs were not suitable for SERS due to the lack of a sub-nm gap (Fig. 5B).^64^ The Raman enhancement factor (EF) of the 37.5 nm dimers was estimated to be 5 × 10^5^ based on the vibration peak at 1021 cm^–1^ (ring breathing mode), according to the definition in the literature (see Fig. S18† for the normal Raman signals of 4-MPy).^65^ The lack of BSPP signals in the Raman spectra could be attributed to the stripping of BSPP by Ag^+^, consistent with the XPS data (Fig. S2 and S3†) and the proposed mechanism in Fig. 1. Such an EF is better called an analytical EF (AEF),^65^ since it was averaged through all dye molecules (adsorbed or not adsorbed) and different dimer orientations relative to the incident laser polarization. Considering the above situation and bearing in mind that only 4-MPy molecules adsorbed in the “hotspots” could give a measurable Raman signal, the 5 × 10^5^ enhancement should be a significant underestimate (several orders lower) of the real EF inside the sub-nm gap.^9^ The as-obtained AEF is also comparable with previous work on well-defined Ag and Au nanodimers.^63^,^66^

To rationalize the strong SERS signals from the 37.5 nm dimers, near-field calculations of all monomeric and dimeric structures were performed based on a discrete dipole approximation (DDA).^67^,^68^ The numerical results in Fig. S19–S22† show that the 37.5 nm dimers had a very strong electric field at the inter-particle gap under 671 nm incident radiation. The electric field at the hottest position (in the middle of two soldered 37.5 nm AuNPs spaced by 0.76 nm) was 4.7 times of that of the 24.1 nm dimer. This corresponds to a 488-fold improvement of the electromagnetic Raman enhancement (|*E*|^4^/|*E*_0_|^4^) for the 37.5 nm dimers compared to the 24.1 nm dimers.^69^ It is therefore easy to understand why the 24.1 nm dimer and all monomers did not give resolvable Raman signals.

## Conclusions

In conclusion, we have developed a novel strategy to produce strongly coupled plasmonic as well as catalytic nanoparticle oligomers by a homogeneous one-pot solution approach based on an Ag^+^ soldering-triggered rapid and self-limiting (Fig. S8 and S23†) colloidal assembly. This process is very simple, highly efficient, cost-effective, and thus suitable for the large scale production of plasmonic and catalytic nanoassemblies. The tunable strong coupling and high structural purity along with the excellent stability of the nanoparticle clusters makes them very attractive toward the fabrication of plasmonic devices, SERS substrates, and nanocatalysts. The structural uniformity and the ease of further surface functionalization makes the clusters excellent candidates as plasmonic and light scattering bio-probes toward cellular sensing, therapy, and imaging. Compared to Au nanorods with a similar structural anisotropy, the gold dimers are interesting due to the presence of a sub-nm metallic gap (ideal for surface enhanced spectroscopy and hot electron-mediated photocatalysis in a plasmonic metal/molecule/metal junction^70^–^72^) and a truly surfactant-free surface (highly desired for biological uses). Therefore, our results will benefit both fundamental and frontier research areas including light scattering, molecular sensing, light harvesting, bio-imaging, and metafluids. Further studies toward the fine engineering of inter-particle gaps and a wide tuning of plasmonic coupling to the near infra-red (NIR) domain are currently being undertaken.

## Supplementary Material

Supplementary movie

Supplementary information
