# Supplementary material for: “Flash” preparation of strongly coupled metal nanoparticle clusters with sub-nm gaps by Ag+ soldering: toward effective plasmonic tuning of solution-assembled nanomaterials
Source: Chem Sci. 2016 May 4;7(8):5435–40. doi: 10.1039/c6sc01407k (PMC6021751; doi:10.1039/c6sc01407k)
Supplement: Supplementary file 2 [file SC-007-C6SC01407K-s002.pdf]

Electronic Supplementary Material (ESI) for Chemical Science.
This journal is © The Royal Society of Chemistry 2016

"Flash" Preparation of Strongly Coupled Metal Nanoparticle Clusters with Sub-nm Gaps by Ag^+ Soldering: Toward Effective Plasmonic Tuning of Solution-Assembled Nanomaterials

Miao Liu,[a] Lingling Fang,[a] Yulin Li,[a] Ming Gong,[b] An Xu[c] and Zhaoxiang Deng*[a]

[a] CAS Key Laboratory of Soft Matter Chemistry & Collaborative Innovation Center of Suzhou Nano Science and Technology, Department of Chemistry, University of Science and Technology of China, Hefei, Anhui 230026, China.
Email: zhxdeng@ustc.edu.cn
[b] Engineering and Materials Science Experiment Center, University of Science and Technology of China, Hefei, Anhui 230027, China
[c] Key Laboratory of Ion Beam Bioengineering, Hefei Institutes of Physical Science, Chinese Academy of Sciences, Hefei, Anhui 230031, China

Experimental Details

Chemicals

Fish sperm DNA (FSDNA), NaCl and $AgNO_3$ were purchased from Bio Basic Inc. (BBI, Canada). Bis(*p*-sulfonatophenyl)phenylphosphine dihydrate dipotassium salt (BSPP) and sodium tetrachloropalladate trihydrate ($Na_2PdCl_4{\cdot}3H_2O$) were obtained from Strem Chemicals (Newburyport, MA, USA). 4-mercaptopyridine was from Acros Organics. Chloroauric acid tetrahydrate ($HAuCl_4{\cdot}4H_2O$) and hexachloroplatinic acid hexahydrate ($H_2PtCl_6{\cdot}6H_2O$) were bought from Sinopharm Chemical Reagent Co., Ltd. (Shanghai, China). Sodium citrate tribasic dihydrate was purchased from Sigma. All reagents were used as received without further purifications.

Nanoparticle synthesis

Citrate-capped AuNPs with diameters of 5.5 and 13.3 nm were synthesized following literature methods.[1-2] Other nanoparticles used in this work were prepared according to the following procedures.

24.1 nm AuNPs[3]

13.3 nm AuNPs were used as nucleation seeds to grow 24.1 nm AuNPs. Immediately after the synthesis of 13.3 nm AuNPs (50 mL), the solution was cooled to 90 °C. 660 μL of sodium citrate (60 mM) and 660 μL of $HAuCl_4$ (25 mM) were sequentially added (time delay of ~2 min between the two additions) to this solution, with the mixture being maintained at 90°C under vigorous stirring for 30 min. By repeating the additions of sodium citrate and $HAuCl_4$ up to 5 times, AuNPs with a diameter of 24.1 nm were obtained.

37.5 nm AuNPs [3]

Immediately after the synthesis of 24.1 nm AuNPs (56 mL), 660 μL of 25 mM $HAuCl_4$ was injected. After an incubation at 90 °C under stirring for 30 min, 30 mL of the reaction solution was extracted, which was then diluted by mixing with 28 mL of deionized water and 2 mL of 60 mM sodium citrate. The above mixture was heated to 90 °C in about 30 min before an injection of 1 mL of 25 mM $HAuCl_4$. The same heating and $HAuCl_4$ addition were repeated one more time, with the final solution being heated at 90 °C for another 30 min to produce 37.5 nm AuNPs.

30 nm PtNPs[4]

The synthesis of 30 nm PtNPs started with a preparation of 5 nm PtNP seeds. Briefly, 46.4 mL of deionized water was heated to boiling before the addition of 72 μL of H_2PtCl_6 (193.1 mM)

under strong stirring. 1 min after adding H_2PtCl_6, 1.1 mL of a solution containing 1% sodium citrate and 0.05% citric acid was added. 0.5 min later, 550 μL of a freshly prepared solution containing sodium borohydride (0.08%), sodium citrate (1%) and citric acid (0.05%) was introduced. The solution was kept boiling for 10 min before being cooled down to room temperature to obtain 5 nm PtNP seeds. To obtain 30 nm PtNPs, 1 mL of the 5 nm PtNP seeds was diluted with 29 mL of deionized water at room temperature. Afterwards, 93.2 μL of H_2PtCl_6 (193.1 mM) and a 0.5 mL solution containing sodium citrate (1%) and L-ascorbic acid (1.25%) were sequentially introduced. The mixture was slowly heated (~10°C/min) to boiling and maintained under this condition for about 40 min to produce 30 nm PtNPs.

29 nm Au@PdNPs[5]

2.47 mL of deionized water was pre-cooled in an ice-water bath. 80.6 μL of BSPP-capped 13.3 nm AuNPs (0.248 μM), 95.4 μL of Na_2PdCl_4 (100 mM), and 123.1 μL of sodium citrate (100 mM) were sequentially added to the pre-cooled water. Afterwards, 1.23 mL of ice-cooled L-ascorbic acid (100 mM) was slowly added dropwise to the above solution under continuous stirring. The reaction lasted 45 min to obtain 29 nm Au@PdNPs.

BSPP ligand exchange

BSPP was added to a freshly prepared nanoparticle solution to reach a concentration of 0.2 mg/mL (for 5.5 and 13.3 nm AuNPs) or 1 mg/mL (for 24.1 and 37.5 nm AuNPs, 30 nm PtNPs, and 29 nm Au@PdNPs). The solution was kept standing at room temperature for 8 h followed by a centrifugation and redispersion of the nanoparticles in deionized water.[6]

Ag^+ triggered self-assembly of discrete nanoparticles

Au, Pt, or Au@Pd nanoparticles were dispersed in 0.5×TBE (Tris, 44.5 mM; EDTA, 1 mM; boric acid, 44.5 mM; pH 8.0) buffer containing 1 mg/mL FSDNA and appropriate amount of $AgNO_3$ (0.5 to 20 mM). The solution was kept at room temperature for a soldering reaction to take place. In the case of AuNPs, the color of the solutions changed from red to purple (or blue) within seconds (see a supporting video clip).

Agarose gel purification of nanoparticle oligomers

A mixture of nanoparticle clusters was loaded in a 3% (for 5.5 and 13.3 nm AuNPs) or 1.5% (for larger AuNPs, PtNPs, and Au@PdNPs) agarose gel, which was run in 0.5×TBE at 13 V/cm for an electrophoretic separation. To recover a desired oligomer, the corresponding gel band was cut out and simply soaked in a 0.5×TBE buffer for several hours to elute the product. If an electric field is applied to accelerate the elution,[6] the whole process including sample preparation, gel separation, and product elution will be finished in1-2 hours.

Ultrasonication-based stability test

Purified AuNP dimers were dispersed in water, followed by a sonication at 12% of the full power (130 W) of a VCX 130PB probe-type sonicator (Sonics & Materials Inc., USA). A sonication probe with a diameter of 3 mm was used.

X-ray Photoelectron Spectroscopic (XPS) characterization

A sample for XPS analysis was prepared by spotting 30 μL of an AuNP solution onto a 1×1

cm^2 square of aluminum foil. The sample was allowed to dry in air. This spotting-and-drying process was repeated three times to ensure enough sample coverage. XPS measurements were carried out on an ESCALAB 250 high performance electron spectrometer using Al Kα (1486.6 eV) radiation.

Samples for XPS analyses

5 nm AuNPs

5 nm AuNPs were used considering their larger surface area which can give stronger XPS signals of surface adsorbed species. 100 μL of BSPP-exchanged 5 nm AuNPs (4 μM) was centrifuged at 21500 g for 60 min. The supernatant was discarded, and the precipitate was re-dissolved in 100 μL water. This process was repeated up to 5 times to remove excess BSPP in the solution. The final AuNP solution was divided into two 100 μL parts for further experiments (one as a control, and the other for an Ag^+ treatment).

Ag^+ and FSDNA treated 5 nm AuNPs

To one part of the above-obtained AuNPs was added 4 μL of 100 mM $AgNO_3$. The resulting mixture was kept at room temperature for up to 30 min before being subjected to a centrifugation at 21500 g for 30 min to remove dissolvable Ag^+-containing species in the supernatant. The precipitate was dispersed in 100 μL of a FSDNA solution (1 mg/mL). After 30 min, the solution was centrifuged at 21500 g for 1 h, with the precipitate being dissolved in 100 μL water. This process was repeated one more time to remove free DNA and other dissolvable species.

TEM characterization

Transmission electron microscopy (TEM) images were taken on a JEM-2100F field emission TEM operated at an electron acceleration voltage of 200 kV. Samples for TEM imaging were prepared on carbon-coated copper grids. The sample droplets were allowed to stay on the TEM grids for 5 minutes before being removed by gently touching with a piece of filter paper.

Spectroscopic characterizations

Optical extinctions were measured on a Hitachi U-2910 UV-Vis spectrophotometer. Raman scattering was measured at room temperature with a low-volume liquid cell (40-100 μL) on a portable Ocean Optics Raman system equipped with a Maya 2000 CCD detector and two incident lasers (532 and 671 nm).

Reference:

[1] Slot, J. W.; Geuze, H. J. *Eur. J. Cell. Biol.* **1985**, 38, 87-93.

[2] Frens, G. *Nat. Phys. Sci.* **1973**, 241, 20-24.

[3] Bastús, N. G.; Comenge, J.; Puntes, V. *Langmuir* **2011**, 27, 11098-11105.

[4] Bigall, N. C.; Härtling, T.; Klose, M.; Simon, P.; Eng, L. M.; Eychmüller, A. *Nano Lett.* **2008**, 8, 4588-4592.

[5] Hu, J. W.; Zhang, Y.; Li, J. F.; Liu, Z.; Ren, B.; Sun, S. G.; Tian, Z. Q.; Lian, T. *Chem. Phy. Lett.* **2005**, 408, 354-359.

[6] Deng, Z. X.; Tian, Y.; Lee, S. H.; Ribbe, A. E.; Mao, C. D. *Angew. Chem. Int. Ed.* **2005**, 44, 3582-3585.

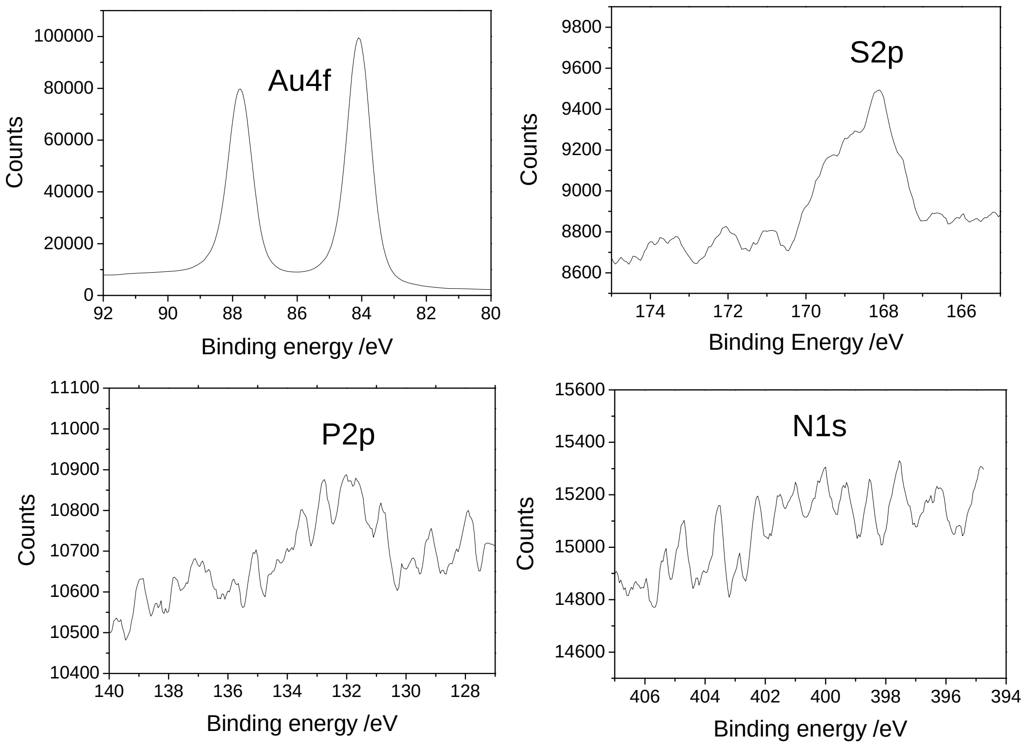

Figure S1. High resolution XPS spectra for Au, S, P, and N elements of BSPP-exchanged 5 nm gold nanoparticles. The S and P elements came from the BSPP ligands on the AuNPs.

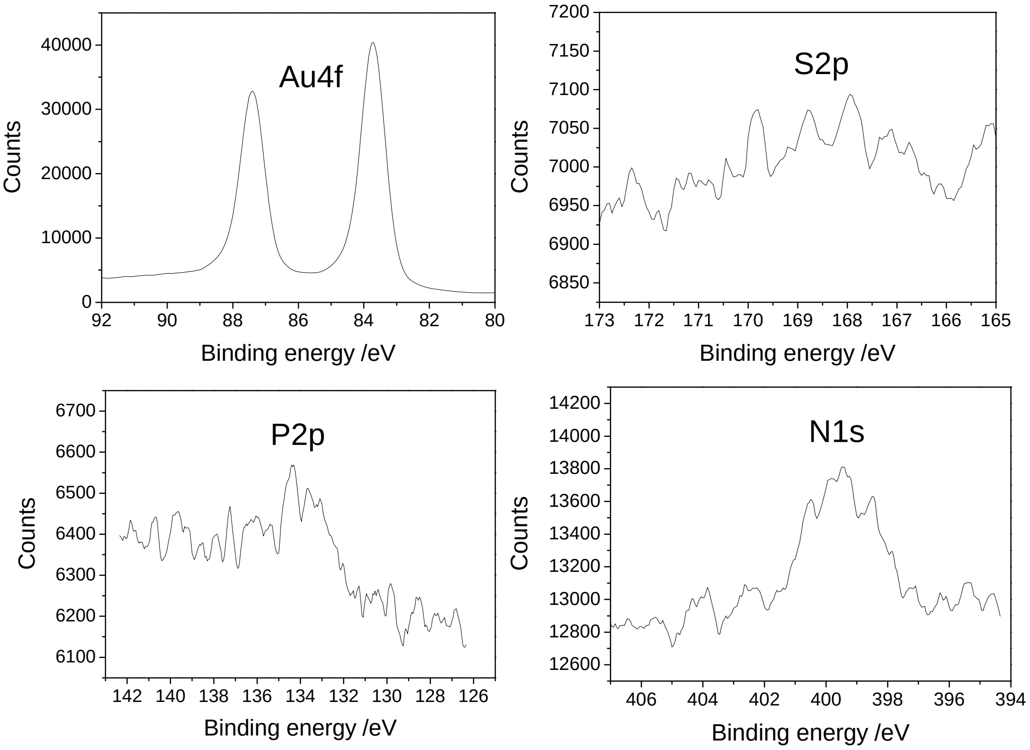

Figure S2. High resolution XPS spectra for Au, S, P, and N elements of BSPP-exchanged 5 nm gold nanoparticles after Ag^+ treatment and FSDNA adsorption. **A decrease of S signal implied a partial desorption of BSPP ligands from the AuNPs. The N signal came from the DNA bases of surface-adsorbed FSDNA on the AuNPs. The P signal could be weakened due to a desorption of BSPP but then enhanced by the phosphate backbone of adsorbed FSDNA** (the overall intensity of the P signal thus did not show a big change).

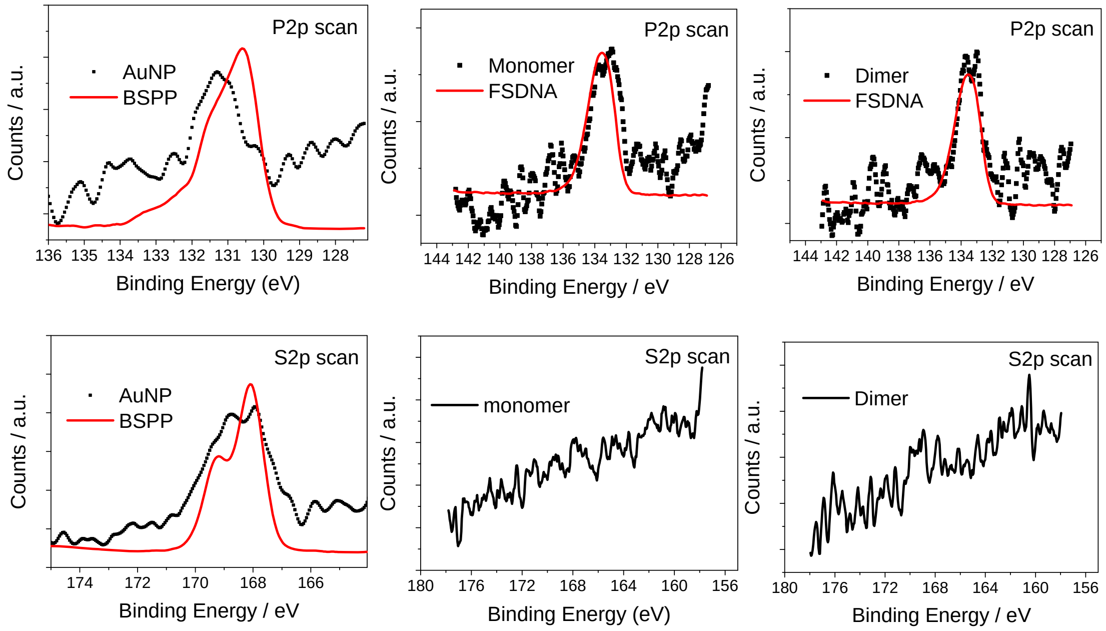

Figure S3. High resolution XPS P2p (upper panels) and S2p (lower panels) spectra of BSPP-exchanged 13 nm AuNPs as well as monomeric and dimeric products isolated from Ag^{+} soldered (in the presence of FSDNA) 13 nm AuNPs by agarose gel electrophoresis. XPS data of BSPP and FSDNA have also been measured for comparisons.

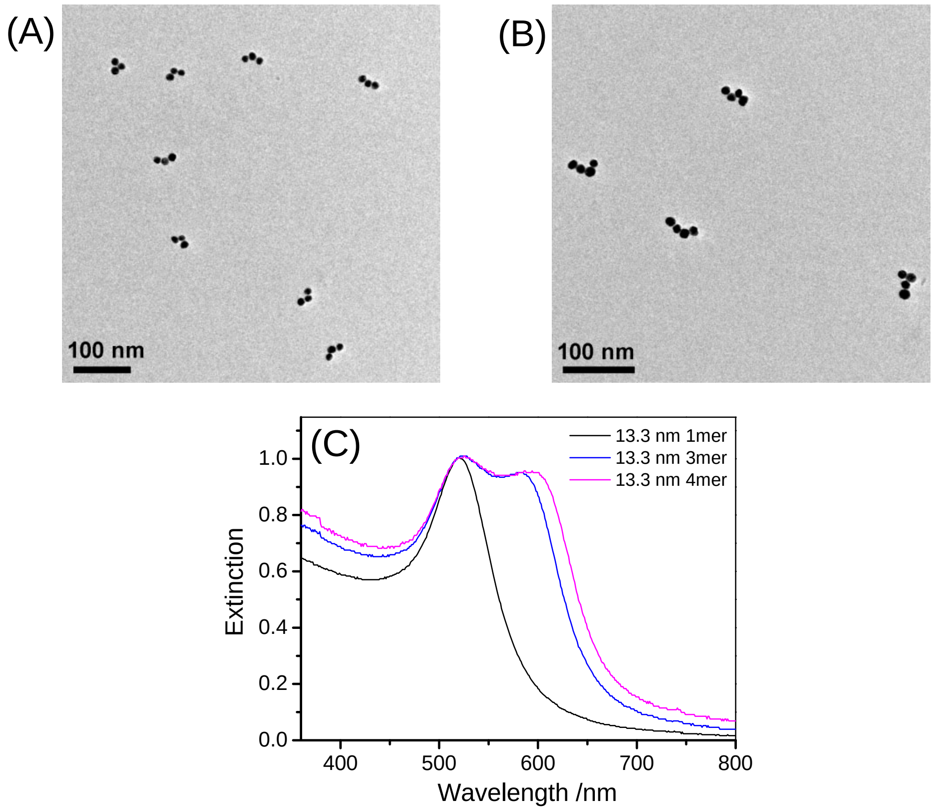

Figure S4. (a,b) TEM images of purified 13.3 nm AuNP trimers and tetramers; (c) Visible extinction spectra of monomeric, trimeric, and tetrameric 13.3 nm AuNPs after Ag^{+} soldering.

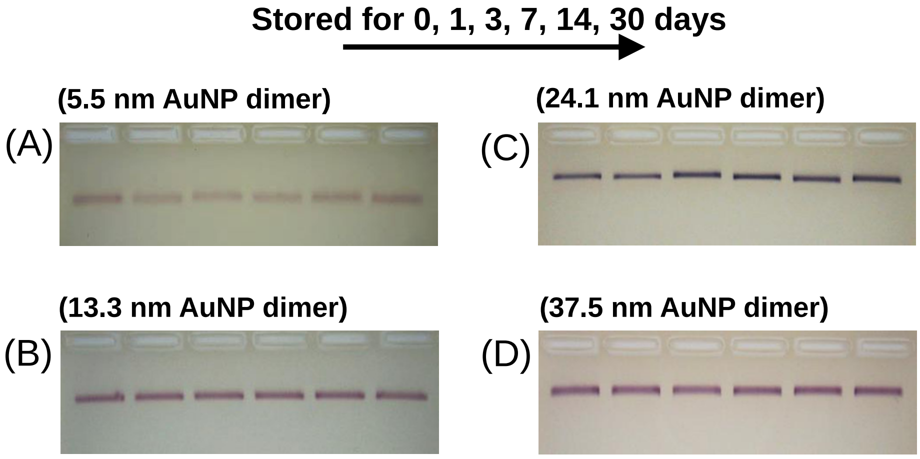

Figure S5. Agarose gel electrophoretic stability examination of 5.5 nm, 13.3 nm, 24.1 nm, and 37.5 nm AuNP dimers after being stored in water at room temperature over one month.

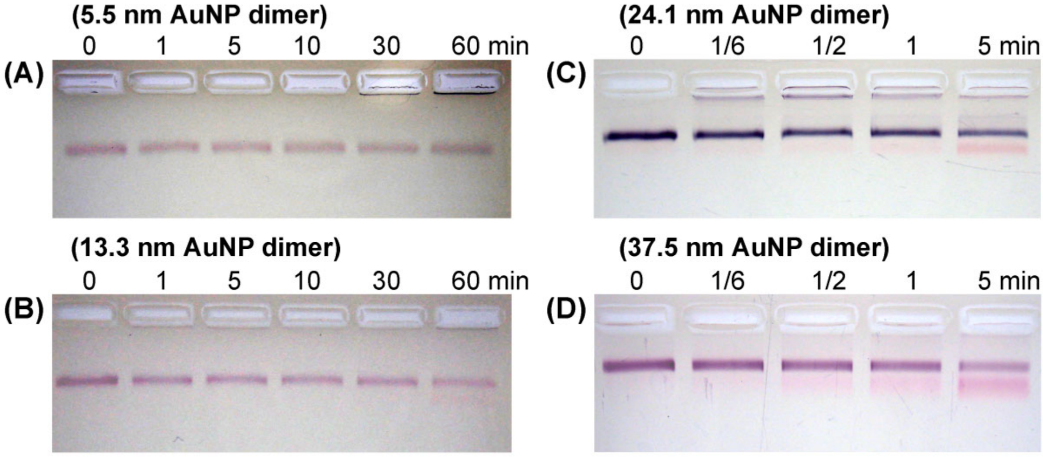

Figure S6. Agarose gel electrophoresis of AuNP dimers treated by strong sonications for different periods. Dimers with a larger size were more susceptible to such a mechanical disruption.

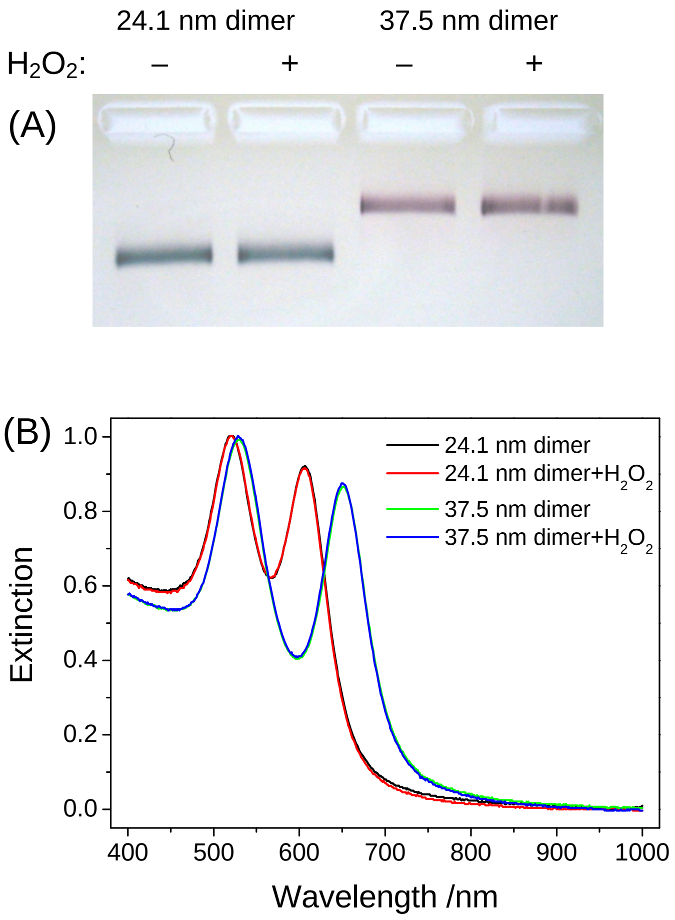

Figure S7. Gel electrophoresis (A) and visible extinction spectra (B) of 24.1 nm and 37.5 nm AuNP dimers after being etched with 10% H_2O_2 for 30 min to remove possibly existing Ag(0) species. No changes were caused by the H_2O_2 treatment.

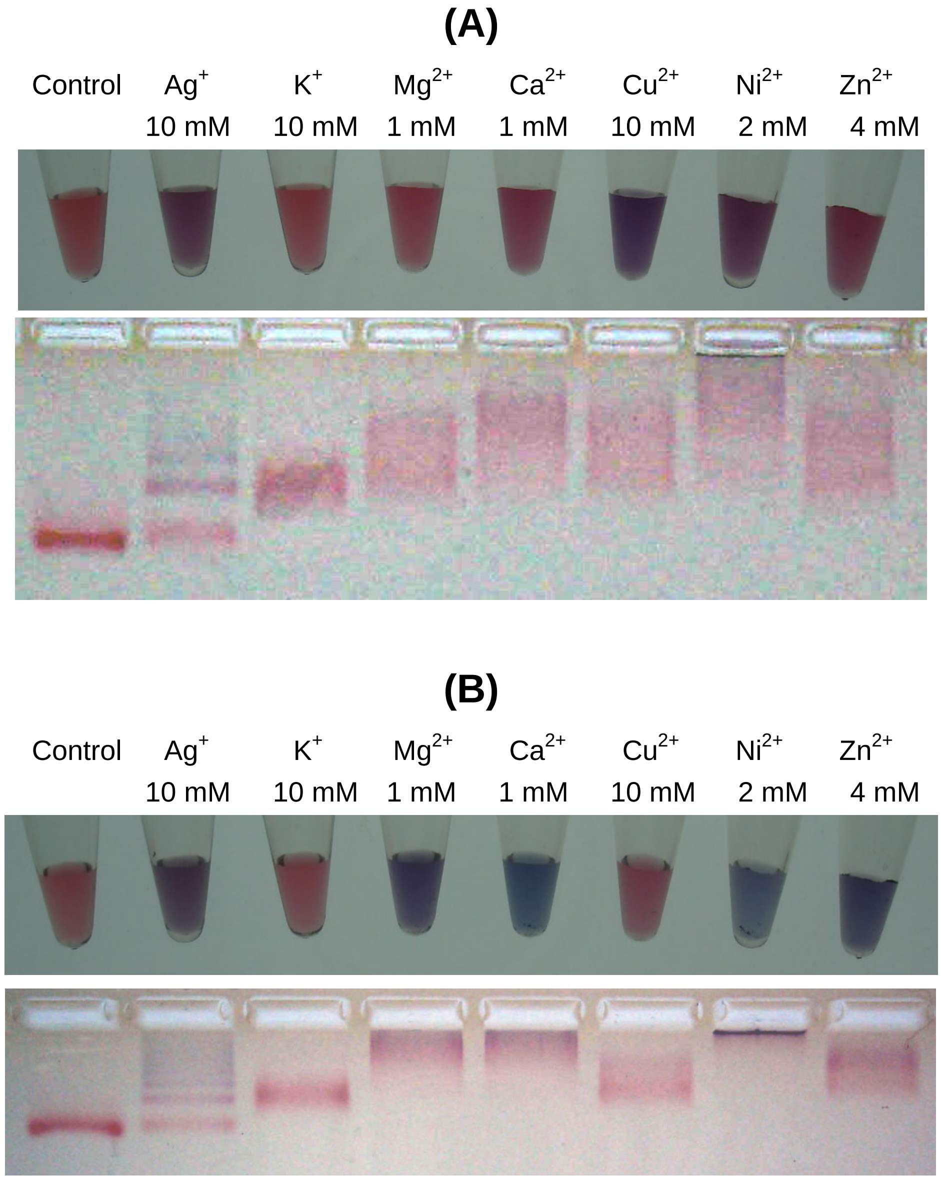

Figure S8. Agarose gel electrophoresis of 13.3 nm AuNPs after being treated by different metal ions in the presence of FSDNA. Samples in (b) corresponded to the same samples in (a) after a precipitation (by centrifugation) and redispersion in water before gel electrophoresis. It is clearly seen that only Ag^{+} treated samples formed stable and sharp gel bands.

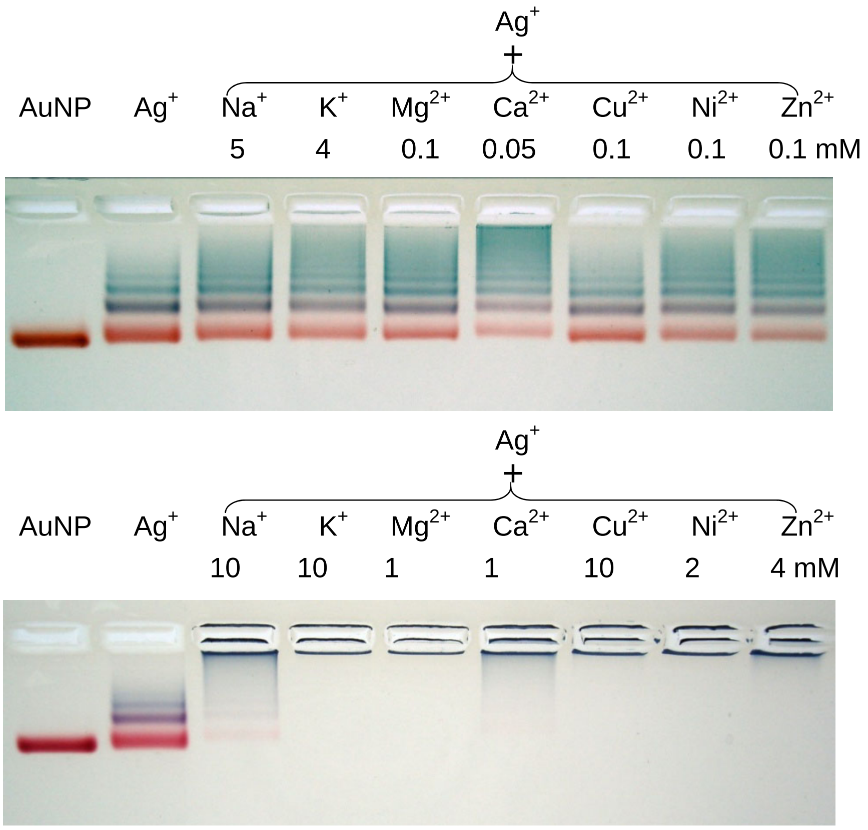

Figure S9. 2% Agarose gel electrophoresis showing that coexisting metal ions at relatively low concentrations (upper panel) promoted Ag^{+} soldering efficiency of AuNPs, while high concentrations of coexisting cations (lower panel) led to large AuNP aggregates that did not enter the gel.

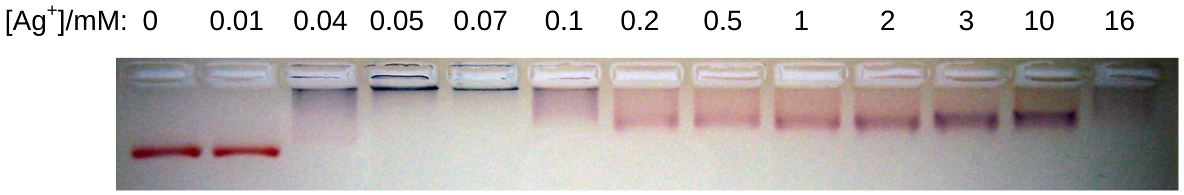

Figure S10. Agarose gel electrophoresis of 13.3 nm AuNPs after being treated by different concentrations of Ag^{+} in the absence of FSDNA.

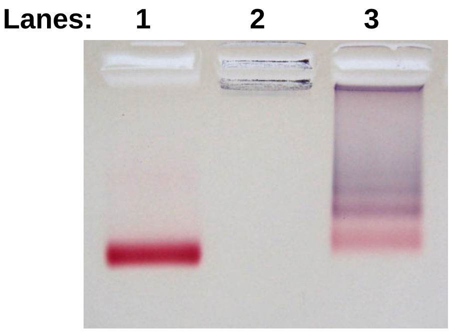

Figure S11. Agarose gel electrophoresis showing that Ag^{+}-induced aggregates (lane 2) of BSPP-capped 13.3 nm AuNPs (lane 1) could be re-dispersed into discrete gel bands (lane 3) after an addition of FSDNA.

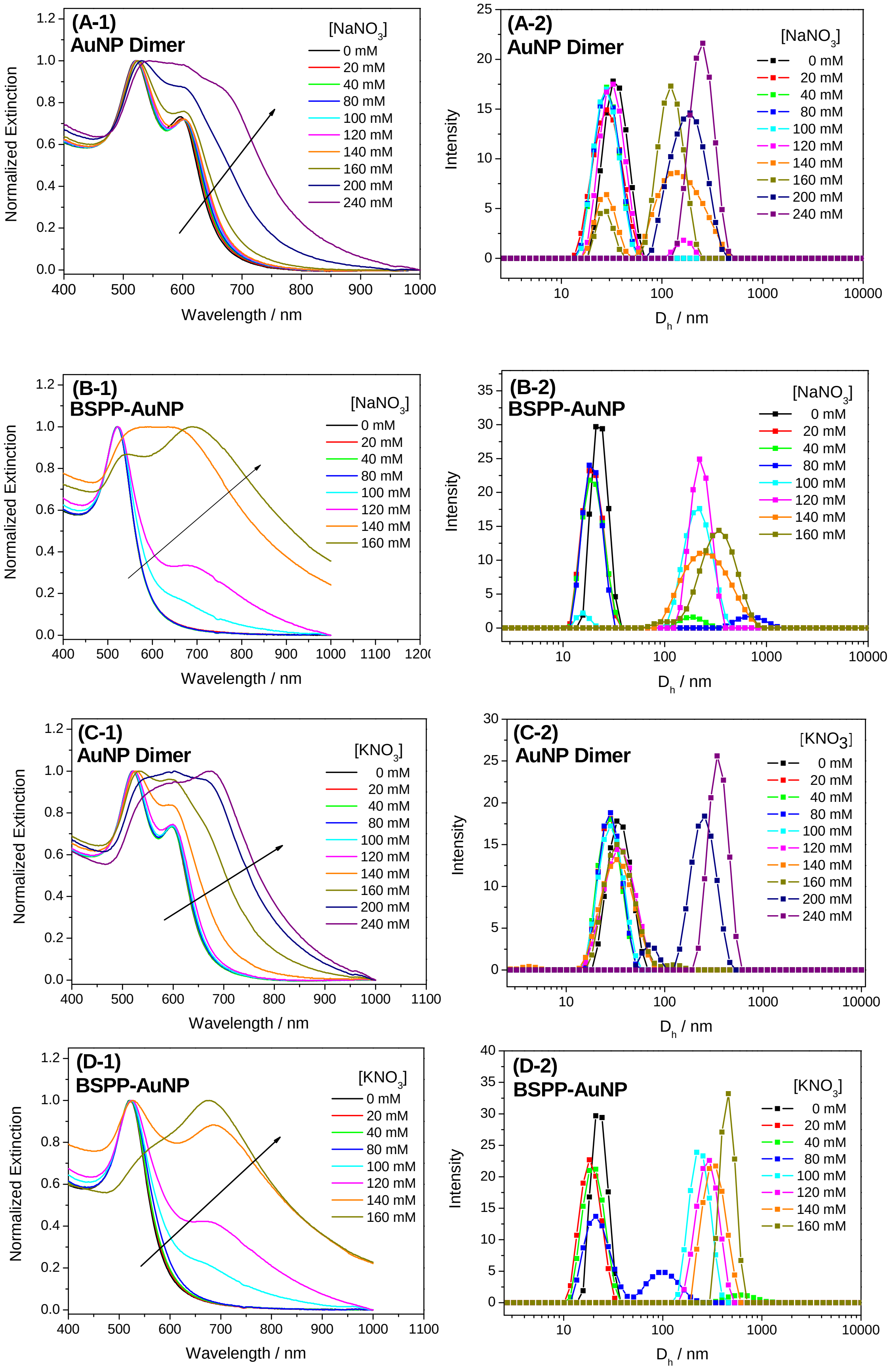

Figure S12. UV-Vis (left panels) and DLS (right panels) data reflecting the salt resistivity of Ag^{+} soldered dimers (A, C) in contrast to BSPP-capped AuNPs (B, D) against Na^{+} (A, B) and K^{+} (C, D). These data revealed an even better stability of the dimer product against Na^{+} and K^{+} due to surface-adsorbed FSDNA.

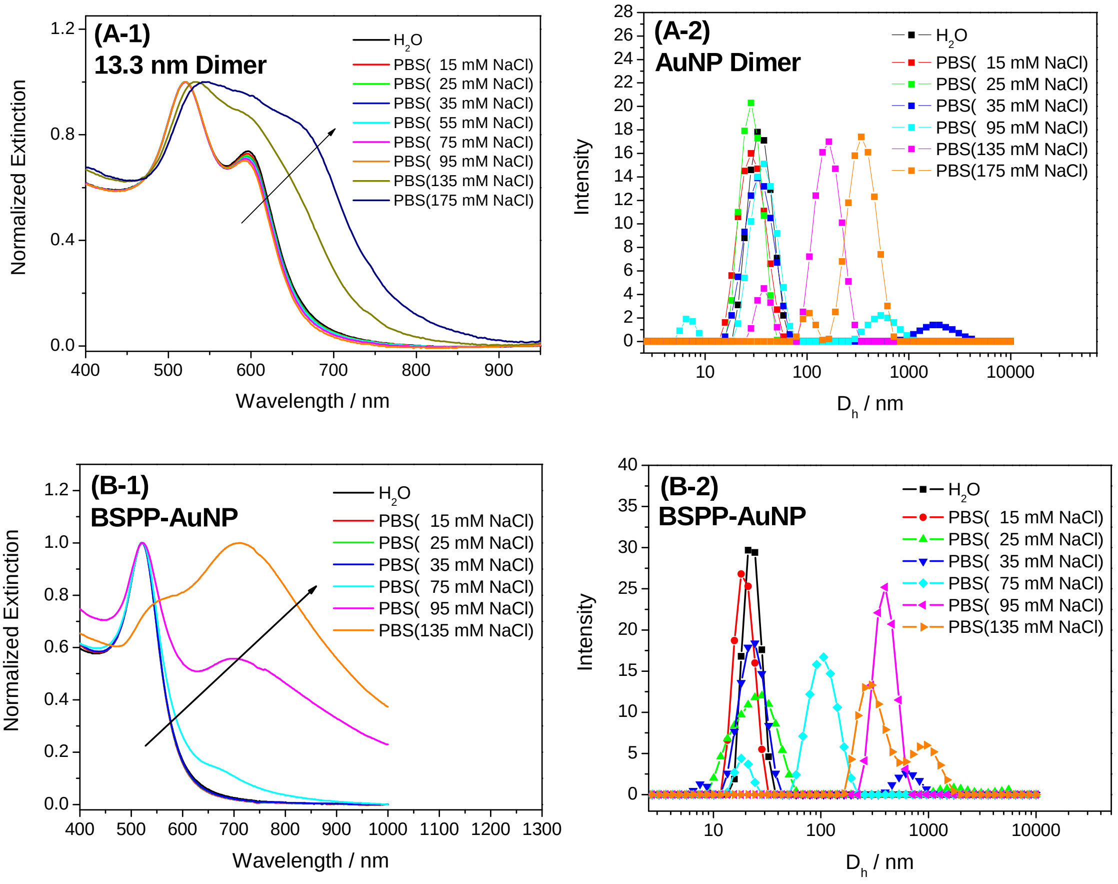

Figure S13. UV-Vis and DLS data reflecting the stability of Ag^+ soldered dimers (A) in comparison with BSPP-capped AuNPs (B) in a PBS buffer (pH 7.4) containing 8.2 mM Na_2HPO_4, 1.8 mM KH_2PO_4, and different amounts of NaCl. These data revealed a much better stability of the dimer product in a saline buffer due to the presence of surface-adsorbed FSDNA.

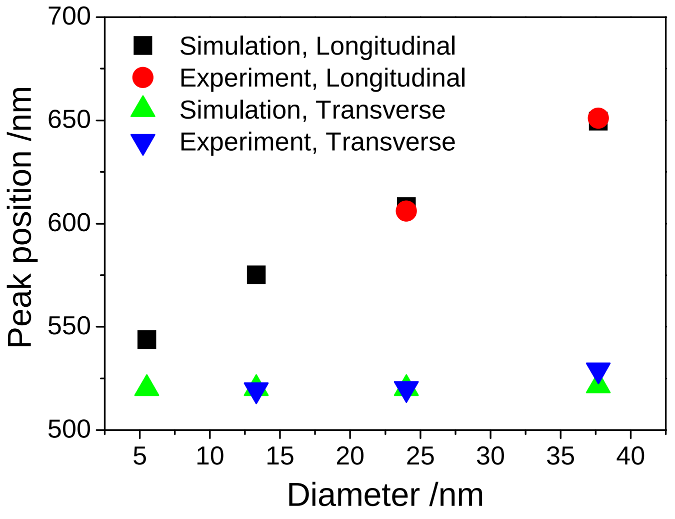

Figure S14. Comparisons of plasmonic peak positions between experiments and simulations for 5.5 nm, 13.3 nm, 24.1 nm, and 37.5 nm AuNP dimers.

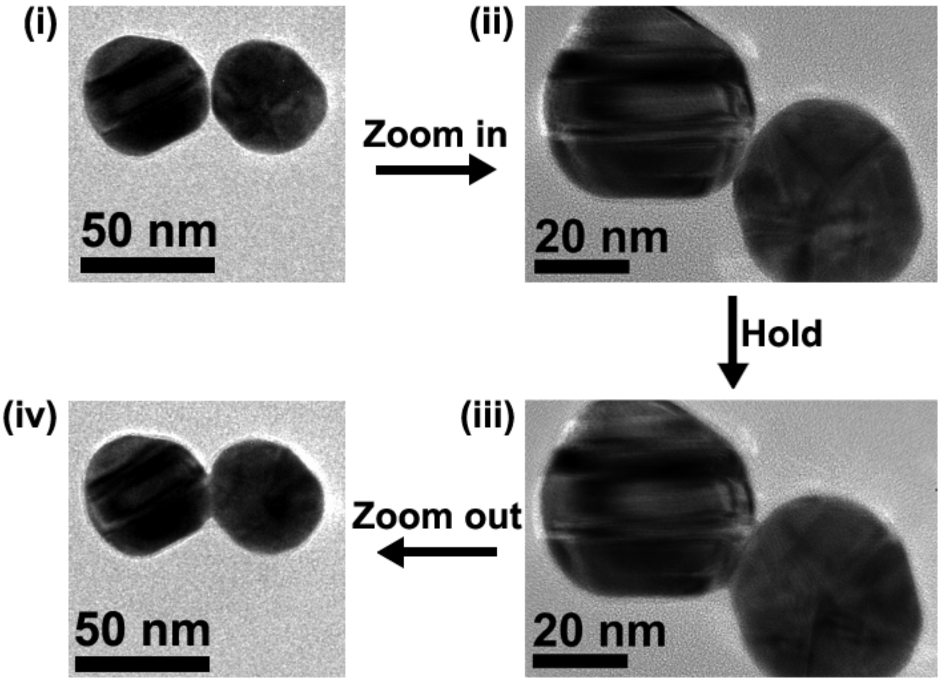

Figure S15. TEM images showing the approaching and coalescence of two AuNPs in a dimer structure under electron beam irradiation in a zoom-in and zoom-out cycle.

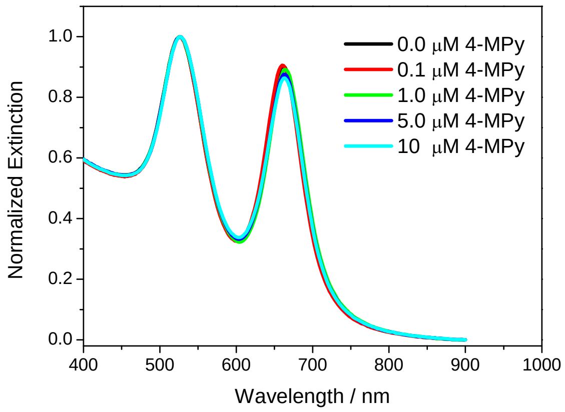

Figure S16. Visible extinction spectra accounting for the good stability of 37.5 nm AuNP dimers in the presence of 4-MPy at concentrations close to that for SERS measurements.

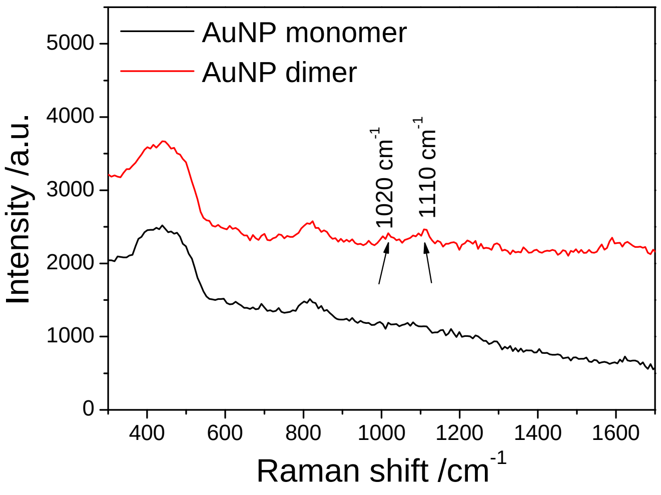

Figure S17. Raman spectra of 4-MPy (10 μM) in the presence of 37.5 nm AuNP monomers and dimers measured with a 532 nm incident laser.

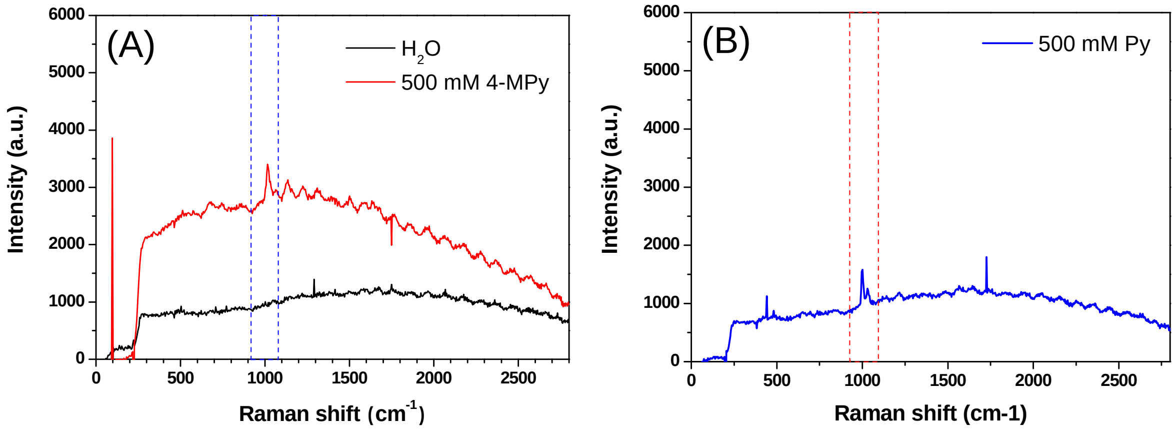

Figure S18. Normal Raman spectra of 0.5 M 4-MPy (A) and pyridine (B) aqueous solutions excited by a 670 nm laser.

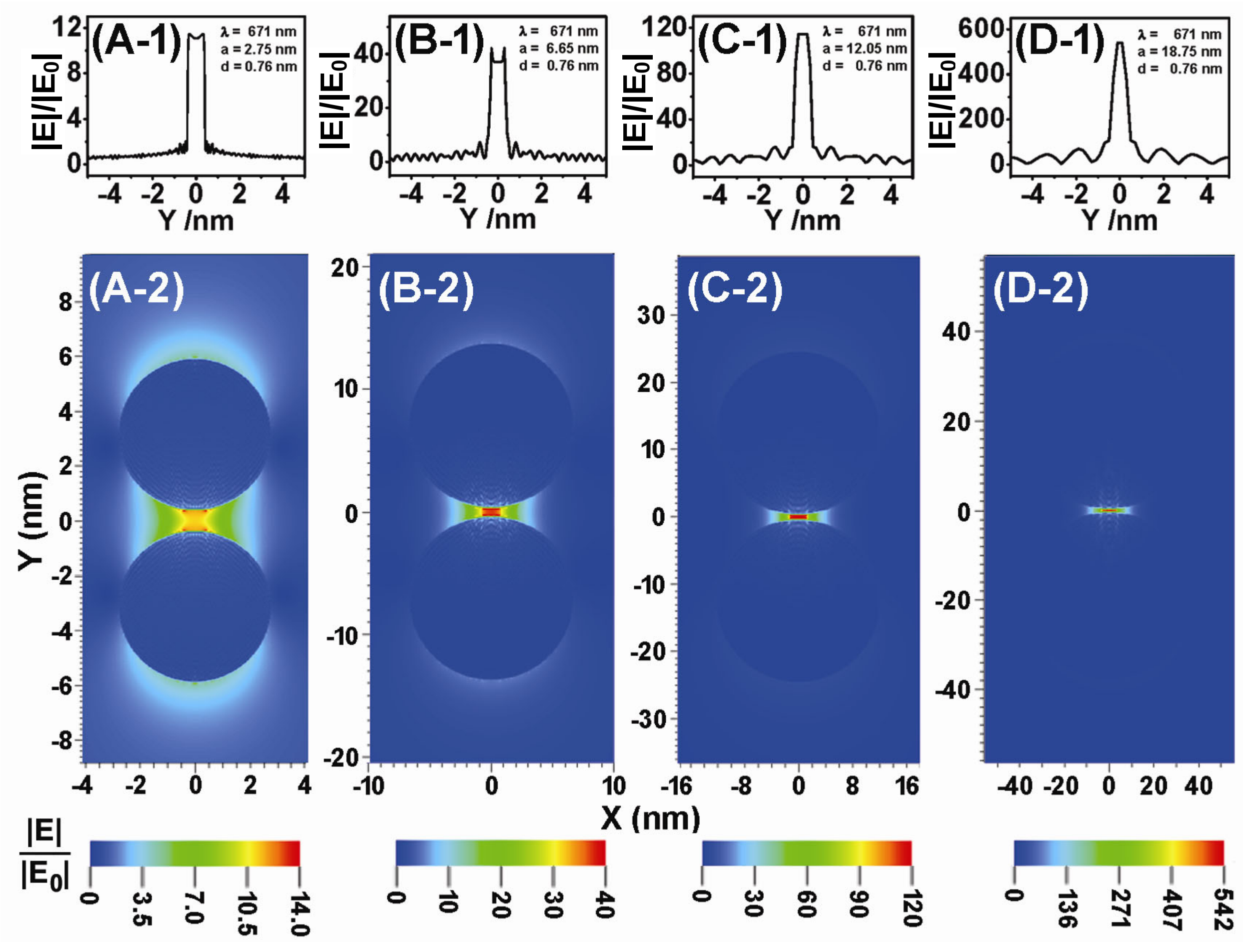

Figure S19. 2D views of electric field distributions for 5.5 (A), 13.3 (B), 24.1 (C), and 37.5 nm (D) gold nanoparticle dimers obtained through near field calculations with DDASCAT 7.3. The top panels show electric field intensity profiles along center-to-center track lines of the dimers. A 671 nm incident radiation polarized along Y axis and propagating along X axis was assumed. Lattice spacing of 2*a*/160 was used for all simulations, where 2*a* represents nanoparticle diameter.

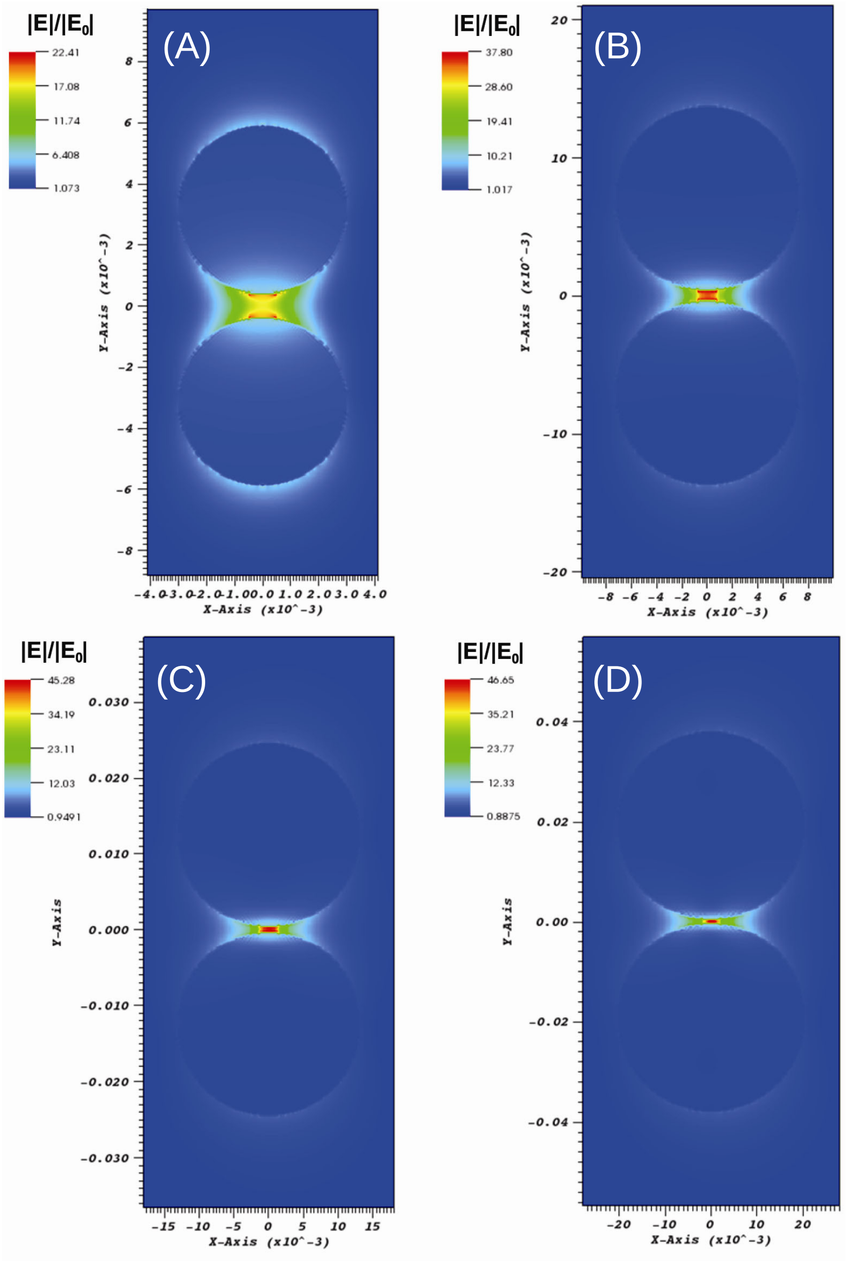

Figure 20. 2D views of electric field distributions for 5.5 (A), 13.3 (B), 24.1 (C), and 37.5 nm (D) gold nanoparticle dimers calculated by DDASCAT 7.3. A 532 nm incident radiation polarized along Y axis and propagating along X axis was assumed.

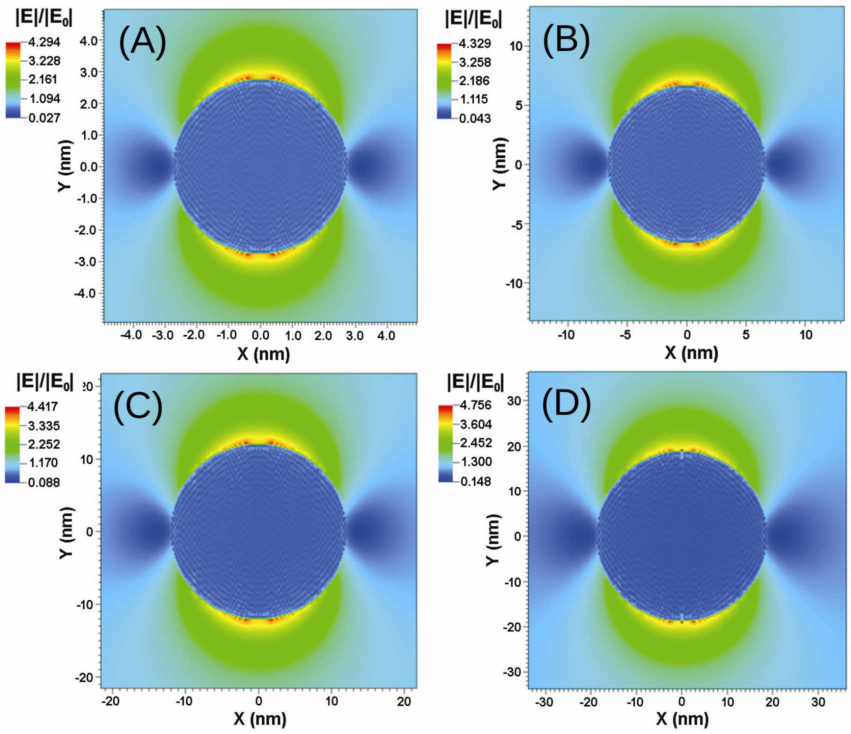

Figure 21. 2D views of electric field distributions for 5.5 (A), 13.3 (B), 24.1 (C), and 37.5 nm (D) gold nanoparticle monomers calculated with DDASCAT 7.3. A 671 nm incident radiation polarized along Y axis and propagating along X axis was assumed.

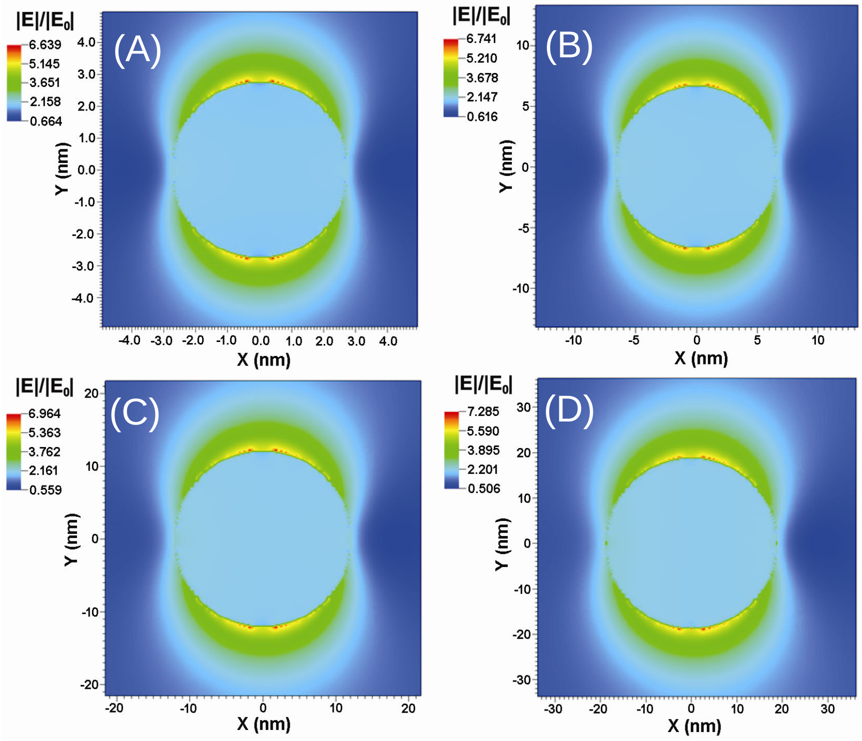

Figure 22. 2D views of electric field distributions for 5.5 (A), 13.3 (B), 24.1 (C), and 37.5 nm (D) gold nanoparticle monomers calculated with DDASCAT 7.3. A 532 nm incident radiation polarized along Y axis and propagating along X axis was assumed.

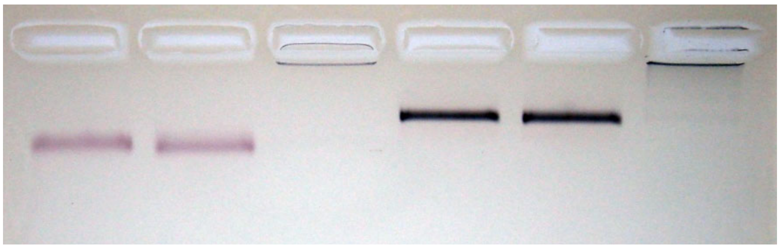

Figure S23. Gel electrophoresis showing that as-purified AuNP dimers were stable in an Ag^+ solution with a concentration equal to that used for Ag^+ soldering. The dimers got further reacted with Ag^+ and precipitated in a 10× concentrated Ag^+ solution.

Table S1. TEM-based statistical analysis indicating a high purify of gel-isolated dimers.

Size/Material	Monomer counts (N_{mo})	Dimer counts (N_{di})	Trimer counts (N_{tr})	Dimer yield [$2N_{di}/(N_{mo}+2N_{di}+3N_{tr})$]
5.5 nm/Au	17	245	3	95%
13.3 nm/Au	6	239	2	98%
24.1 nm/Au	19	229	3	94%
37.5 nm/Au	19	243	6	93%
29 nm/Au@Pd	23	226	7	91%
30 nm/Pt	10	204	1	97%
